# Individualized Template MRI Is a Valid and Reliable Alternative to Individual MRI for Spatial Tracking in Navigated TMS Studies in Healthy Subjects

**DOI:** 10.3389/fnhum.2020.00174

**Published:** 2020-05-14

**Authors:** Robert Fleischmann, Arvid Köhn, Steffi Tränkner, Stephan A. Brandt, Sein Schmidt

**Affiliations:** ^1^Vision and Motor System Research Group, Department of Neurology, Charité – Universitätsmedizin Berlin, Berlin, Germany; ^2^Department of Neurology, University Medicine Greifswald, Greifswald, Germany

**Keywords:** transcranial magnetic stimulation, navigation, spatial transformation, primary motor cortex, motor evoked potentials, template magnetic resonance images

## Abstract

**Objectives**: Navigated transcranial magnetic stimulation (nTMS) provides significant benefits over classic TMS. Yet, the acquisition of individual structural magnetic resonance images (MRI_individual_) is a time-consuming, expensive, and not feasible prerequisite in all subjects for spatial tracking and anatomical guidance in nTMS studies. We hypothesize that spatial transformation can be used to adjust MRI templates to individual head shapes (MRI_warped_) and that TMS parameters do not differ between nTMS using MRI_individual_ or MRI_warped_.

**Materials and Methods**: Twenty identical TMS sessions, each including four different navigation conditions, were conducted in 10 healthy subjects (one female, 27.4 ± 3.8 years), i.e., twice per subject by two researchers to additionally assess interrater reliabilities. MRI_individual_ were acquired for all subjects. MRI_warped_ were obtained through the spatial transformation of a template MRI following a 5-, 9-and 36-point head surface registration (MRI_warped_5_, MRI_warped_9_, MRI_warped_36_). Stimulation hotspot locations, resting motor threshold (RMT), 500 μV motor threshold (500 μV-MT), and mean absolute motor evoked potential difference (MAD) of primary motor cortex (M1) examinations were compared between nTMS using either MRI_warped_ variants or MRI_individual_ and non-navigated TMS.

**Results**: M1 hotspots were spatially consistent between MRI_individual_ and MRI_warped_36_ (insignificant deviation by 4.79 ± 2.62 mm). MEP thresholds and variance were also equivalent between MRI_individual_ and MRI_warped_36_ with mean differences of RMT by −0.05 ± 2.28% maximum stimulator output (%MSO; *t*_(19)_ = −0.09, *p* = 0.923), 500 μV-MT by −0.15 ± 1.63%MSO (*t*_(19)_ = −0.41, *p* = 0.686) and MAD by 70.5 ± 214.38 μV (*t*_(19)_ = 1.47, *p* = 0.158). Intraclass correlations (ICC) of motor thresholds were between 0.88 and 0.97.

**Conclusions**: NTMS examinations of M1 yield equivalent topographical and functional results using MRI_individual_ and MRI_warped_ if a sufficient number of registration points are used.

## Introduction

Navigation systems are increasingly used in research and clinical studies to direct transcranial magnetic stimulation (TMS) induced effects to specific target sites in healthy subjects and patients (Ruohonen and Karhu, 2010; Rothwell, 2012; Fleischmann et al., 2013). These systems use two-dimensional structural images, mostly magnetic resonance images (MRI), of a subject’s head to render an individual three-dimensional head model. This model is subsequently co-registered with the actual shape and position of the head to provide the user with real-time navigation information during TMS sessions (Noirhomme et al., 2004). This navigation information conceptually contains two different subsets of data. On one hand, it provides spatial information about relative positions of the stimulation coil and the participant’s head (subsequently referred to as *spatial tracking*). On the other hand, imaging data includes anatomic information that provides landmarks used to guide the stimulation (subsequently referred to as *anatomical guidance*). While spatial tracking and anatomical guidance are rarely examined separately concerning their contribution to navigated TMS (nTMS) examination quality, their combination certainly provides substantial benefits over non-navigated conditions such as stable coil positioning, orientation, and tilting throughout nTMS examinations (Hannula et al., 2005). A recent study also showed that estimates of cortical excitability are significantly less confounded by small fluctuations of physical covariates in navigated as compared to non-navigated conditions (Schmidt et al., 2015). Another study reported that motor evoked potentials (MEP) elicited by nTMS were more stable, had shorter latencies and larger amplitudes as compared to non-navigated conditions (Julkunen et al., 2009). These findings show that the validity of cortical excitability estimates obtained by nTMS is superior to that of non-navigated systems. One study found that topographical and functional measures obtained by novice and expert users were in good to excellent agreement, indicating that the method is also objective (Fleischmann et al., 2013). The intersession and retest reliability are also reported to be excellent (Hannula et al., 2005; Fleischmann et al., 2013). In summary, nTMS systems should be preferred over non-navigated TMS systems whenever possible.

There are yet constraints in the use of nTMS that hinder a more widespread application of the technology. Important drawbacks are the infrastructure and costs associated with its operation. These drawbacks are substantially dependent on the prerequisite to acquire individual structural MRI before each nTMS exam. Additionally, the image acquisition procedure is time-consuming and not feasible in all subjects, e.g., due to claustrophobia. This being said, it is advisable to reconsider if an individual MRI is inevitably required to perform valid nTMS examinations. To be more precise, possibly, either spatial navigation and/or anatomical guidance are not required at all times. Especially the necessity of anatomical guidance is challenged in M1 that provides a direct read-out of the stimulation location through motor evoked potential (MEP) amplitudes and latencies (Ahdab et al., 2010; Danner et al., 2012). The M1 stimulation hotspot has a functional definition, this being the location where MEP can be elicited with the least stimulator output intensity (Ilić and Ziemann, 2005). In other words, while anatomical guidance facilitates hotspot identification in M1, the lack of anatomical information can by definition not challenge the validity of hotspots identified using only neurophysiological criteria (Yousry et al., 1997; Siebner et al., 2009). This leads to the hypothesis that the superiority of nTMS in the assessment and re-assessment of M1 cortical excitability is possibly due to spatial navigation and not anatomical guidance. In this case, nTMS examinations of M1 would not necessarily require an individual MRI but rather any MRI with a matching head shape that provides relative co-registration. Carducci et al. have already proven that such an individualization of a template MRI using external landmarks is possible (Carducci and Brusco, 2012). Unfortunately, the authors did not report how exactly the spatial transformation of the template image was achieved and did not concurrently assess functional data to prove the equivalence of neurophysiological estimates e.g., of cortico-spinal excitability.

This study aimed to test the hypothesis that topographic results and estimates of M1 corticospinal excitability are equivalent between nTMS using an individual MRI vs. an individualized template MRI approach. Since results should be applicable for a wide range of users and independent of user experience, we investigated the interrater reliability of outcome variables between an inexperienced and expert nTMS user.

## Materials and Methods

All procedures involving human participants were conducted following the ethical standards of the institutional research committee and with the 1964 Helsinki declaration and its later amendments. Formal consent from the institutional review board was obtained.

### Participants

Ten healthy subjects volunteered to participate in this study (one female, 27.4 ± 3.8 years old, all right-handed). All subjects gave written informed consent before any data was obtained and participants were free to withdraw without reason at any time. Handedness was confirmed by the Edinburgh handedness inventory. A detailed medical history was taken to exclude neurological or psychiatric illness and the presence of implanted electronic devices or ferromagnetic metals.

The study was conducted by two researchers, one researcher with less than 1-year experience with nTMS and one expert researchers with a 10-year experience with nTMS.

### Design

The study was conducted in a crossover design with two experimental sessions per subject. Sessions were at least 1 week apart and identical for one subject except for the researcher who conducted the experiment, i.e., subjects underwent identical TMS sessions performed by either of the researchers in a random order. Each session included the assessment of identical TMS parameters in five different navigation conditions. These navigation conditions included nTMS using either individual MRI (reference, MRI_individual_), three differently created individualized templated MRI (MRI_warped_), and non-navigated “blind” TMS. The order of the navigation conditions was randomized between subjects.

### Acquisition of Individual MRI and Warping of Individualized MRI

Individual anatomical MRI (3D-MPRAGE, matrix 256 × 256, 180 sagittal slices, voxel size 1 mm^3^, on GE 3 Telsa scanner) were acquired for all subjects participating in the study and served as state-of-the-art input to the navigation system’s software for the creation of an individual head and volume conductor model (hereafter simply referred to as head model).

**Figure 1:**
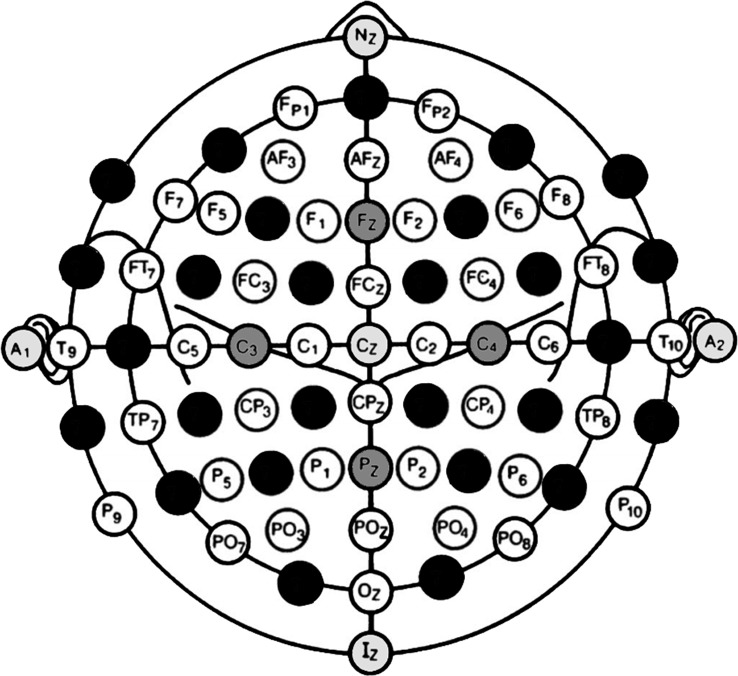
Sensor locations of the 10–10 EEG systems used to warp template magnetic resonanceimages (MRI) to individual head shapes. This study compared topographical and functional results from navigated transcranial magnetic stimulation (TMS) examinations under different navigation conditions. Researchers either used the standard procedure in which navigation was based on participants’ individual MRI or they used template MRI that was adjusted to the subject’s head shape through spatial transformation. Three different conditions included an increasing number of head shape coordinates used for the optimization of the transformation matrix required to adjust the template MRI. The first condition included five sensor locations, which were A1, A2, Nz, Cz, Iz (light gray circles). The second condition was defined by nine sensor locations that comprised the five coordinates of the first condition and another four coordinates (Fz, Pz, C3, C4; dark gray circles). The last condition included 36 equally distributed sensor locations and thus another 27 coordinates were mapped (black circles).

Individualization of template MRI was performed in a two-step procedure. First, three-dimensional surface coordinates of the participant’s head were registered with the navigation system’s digitization pen (eXimia TMS, Nexstim, Helsinki, Finland) and digitally stored in an nx3 matrix (M_n_). Three different conditions included increasing numbers of registered head surface coordinates defined by electrode positions according to the 10-10 EEG system (Acharya et al., 2016). The first condition (“5 points,” M_5_) included three fiducial coordinates (nasion, left and right preauricular), Cz, and Iz coordinates. The second condition (“9 points,” M_9_) included all M_5_ coordinates and additionally Fz, Pz, C3, and C4. In the last condition (“36 points,” M_36_), another 27 coordinates were registered as illustrated in Figure 1.

The second step of the procedure consisted of warping a publicly available T1-weighted high-resolution anatomical template MRI into registered head coordinates (Lalys et al., 2010). To obtain a suitable homogeneous transformation matrix for this operation, the freely available Statistical Parametric Mapping (SPM) and Fieldtrip toolboxes for MATLAB (version 2016a, Mathworks, Gatwick, MA, USA) were used. The names of functions used in this study are provided in italic letters. The registered head coordinates were first brought to the same head coordinate system as the template MRI (i.e., SPM; using *ft*_head coordinates) and the transformation matrix for this operation was stored (T_1_). The registration coordinates M_n_ were then projected onto the scalp surface of the template MRI using *ft*_electrode realign, which returned an equally sized matrix of projected head surface coordinates (pM_n_). An unconstrained nonlinear optimization method was then used to estimate a homogeneous transformation matrix (T_2_) that minimizes the warping error between M_n_ and pM_n_. To be more precise, T_2_ was the local minimum solution of MATLAB’s *fminunc* function, which was set to minimize the result of *ft*_warp_error with M_n_ and pM_n_ as input. The transformation matrix of the template MRI (T_MRI_) was then updated using *ft*_warp_apply that applied a homogenous transformation with given following equation: T_MRI_updated_ = T_2_*T_1_*T_MRI_. The resulting individualized MRI was finally written to a file. This was done for all registered coordinates of one subject and resulted in three individualized MRI (MRI_warped_5_, MRI_warped_9_, MRI_warped_36_). Scripts used in this study are available from the authors upon reasonable request.

### Navigated Transcranial Magnetic Stimulation

The head was tracked by an infrared-based stereotactical system and brought into co-registration with the head model using a triangular system of fiducial coordinates as well as a subsequent nine point surface registration (eXimia TMS, Nexstim, Helsinki, Finland). The registration procedure was also performed in the non-navigated condition using MRI_individual_ to record the mapping procedure and hotspot location but the screen was kept switched off. The number of registration attempts required to perform the registration and the registration error was noted for all but the non-navigated condition. Unsuccessful co-registration attempts included spatial errors larger than 4 mm either based on fiducial registration alone or when aligning fiducial with surface registration points, which resulted in a rejection of the registration attempt and the procedure had to be repeated (Ruohonen and Karhu, 2010).

TMS pulses were delivered by an eXimia TMS stimulator through a biphasic figure-of-eight coil with an outer diameter of 70 mm (Nexstim, Helsinki, Finland). Subjects were seated in a comfortable reclining chair. They were instructed to relax, with their eyes open. Surface EMG-electrodes (Neuroline 700, Ambu, Ballerup, Denmark) were attached to the abductor pollicis brevis (APB), first dorsal interosseus (FDI) and abductor digiti mini (ADM) muscle contralateral to the dominant hemisphere.

Each session performed by one of the researchers consisted of identical successive TMS examinations. First, the dominant hemisphere was mapped for an FDI hotspot. Once a hotspot was identified, the resting motor (RMT) and 500 μV threshold (500 μV-MT) were defined within a 95% confidence interval by an efficient maximum-likelihood threshold algorithm (Awiszus, 2003). Thirty stimuli were then applied at 500 μV-MT intensity over the primary motor cortex hotspot to assess the MEP variance of the targeted neuronal assembly. Equal MEP variances between conditions would provide indirect evidence that equal neuronal assemblies were identified and being examined. MEP variance was defined by the mean absolute difference (MAD) of applied stimuli. MAD was chosen instead of standard deviation since it is a more robust measure of dispersion when underlying data is not normally distributed, which is well known for MEP data at least for an initial transient state of about 20 stimuli (Schmidt et al., 2009).

### Statistics

The feasibility of using MRI_warped_ was evaluated by comparing registration errors and attempts associated with the co-registration procedure for each of the MRI types. The global effects of MRI types on either of the registration parameters were tested with an analysis of variance (ANOVA). Significant global effects were elaborated on by *post hoc* tests including a Bonferroni correction.

Spatial consistency was defined by the Euclidean distance of either MRI_warped_ or non-navigated TMS hotspots to the hotspot identified in the state-of-the-art navigated condition (MRI_individual_). A full model ANOVA was calculated with the main effects *MRI type* and *examiner* to evaluate whether spatial consistency differed between MRI types or examiners.

The functional equivalence of TMS parameters between navigation conditions was assessed by Bland-Altman (BA) plots, which are widely used to assess the agreement between two quantitative methods of measurement (Bland and Altman, 1986). Methods are by definition considered equivalent if: (1) there is no outlier data beyond limits of agreement; (2) paired estimates between methods are not significantly different (i.e., there is no fixed bias); and (3) that the methods agree equally throughout the range of measurements (i.e., there is no proportional bias; Giavarina, 2015). Outlier data was determined by simple descriptive statistics, the presence of a fixed bias was evaluated by two-tailed paired *t*-tests and regression analyses were used to assess whether the difference of methods changed proportionally with increasing values, i.e., if there was a proportional bias. Interrater reliabilities were calculated by two-way mixed intraclass correlations (ICC) for the absolute agreement of single measures.

SPSS^®^ Statistics (version 23, IBM Corporation, Armonk, NY, USA) was used to run all but Bland-Altmann analyses for which we used MATLAB (version 2016a, Mathworks, Gatwick, MA, USA). Group data are reported as mean with its standard deviation and a precision of two decimal places throughout. ICC results are reported with their 95% confidence intervals in brackets. *P*-values are rounded to three decimal places and values lower than 0.001 are not reported exact but as <0.001.

## Results

### Feasibility and Accuracy of nTMS With Individualized MRI

The mean spatial transformation error of projected (pM_n_; ideal transformation) and warped (wM_n_; achieved transformation) head surface coordinates was 0.63 ± 0.46 mm in the M_5_ condition, 2.46 ± 0.54 mm in the M_9_ condition and 2.86 ± 0.5 mm in the M_36_ condition, i.e., an agreement of head model and surface coordinates is easier achieved for a few remote than more nearby coordinates.

A co-registration of the head model and actual head shape succeeded in all cases and with all MRI types. There was a significant global effect of the MRI type used for co-registration on the number of attempts required for a successful co-registration (*F*_(3,76)_ = 39.86, *p* = <0001). Co-registrations with MRI_individual_ and MRI_warped_36_ did not differ significantly and required 1.1 ± 0.31 and 1.15 ± 0.49 attempts, respectively (*p* = 1.00). Significantly more attempts were required in MRI_warped_5_ (4.0 ± 1.17, *p* < 0.001) and MRI_warped_9_ (3.15 ± 1.6, *p* < 0.001) conditions.

There was also a significant global effect of the MRI type used for co-registration on the registration error (*F*_(3,76)_ = 5.1, *p* = 0.003). *Post hoc* analyses revealed no significant difference between MRI_individual_ (3.1 ± 0.79 mm) and MRI_warped_5_ (3.5 ± 0.69 mm, *p* = 0.42) or MRI_warped_36_ (2.95 ± 0.76 mm, *p* = 1.00). Co-registrations with head models based on MRI_warped_9_ were associated with significantly larger spatial errors (3.7 ± 0.47 mm, *p* = 0.044).

### Spatial Consistency of Hotspots Between Navigation Conditions

A stimulation hotspot could be identified in all subjects and conditions. The test for global effects revealed that hotspot distances between experimental conditions and the reference condition (MRI_individual_) significantly differed between conditions (*F*_(3,76)_ = 119.73, *p* = 0.003). The interaction of condition and examiner on hotspot distances was not significant, i.e., differences between examiners (novice vs. expert) were not a significant predictor of hotspot distances (*F*_(3,76)_ = 9.11, *p* = 0.765). *Post hoc* testing revealed that the Euclidean distance to MRI_individual_ hotspots was shortest for MRI_warped_36_ hotspots with a mean distance of 4.79 ± 2.62 mm (range: 1.68–9.62 mm). The second-best approximation was given in the non-navigated condition with a mean distance of 7.78 ± 5.61 mm (range: 0.77–20.01 mm). MRI_warped_5_ and MRI_warped_9_ hotspots were located 9.14 ± 4.46 mm (range: 1.92–19.97 mm) and 10.51 ± 5.65 mm (range: 2.64–13.83 mm) distant from MRI_individual_ hotspots.

### Functional Equivalence of TMS Parameters

**Figure 2:**
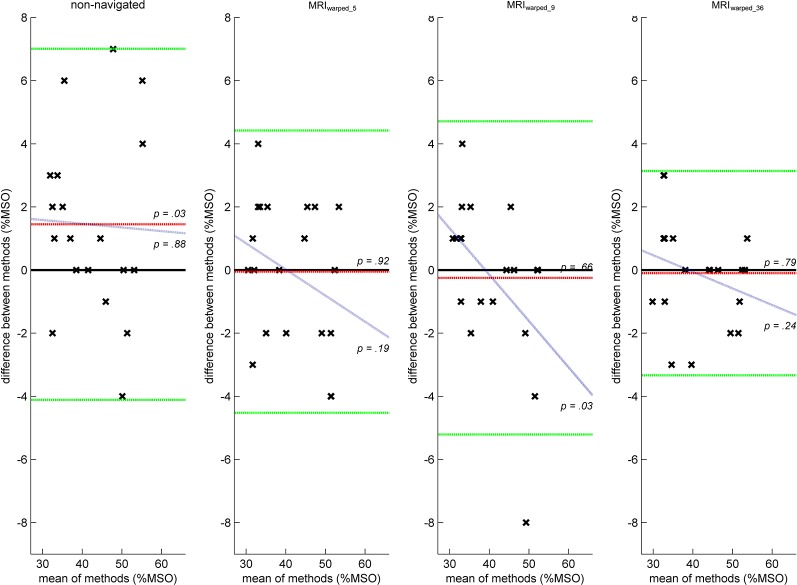
Bland-Altman plots illustrating the agreement of resting motor threshold (RMT) estimates between conditions. Positions of data points (x) represent the absolute differences of RMT estimates (y-axis) plotted against the mean RMT estimates (x-axis) of two different methods assessed twice in 10 subjects. RMT estimates assessed by navigated transcranial magnetic stimulation (nTMS) with individual anatomical MRI (MRI_individual_) were considered reference values and compared to either RMT estimates assessed by nTMS with individualized MRI following a 5-, 9- or 36-point head surface registration (MRI_warped_5_, MRI_warped_9_, MRI_warped_36_) or non-navigated TMS. Ideal agreements of methods would result in differences of 0%MSO irrespective of the RMT magnitude. Red lines indicate the mean difference between methods and right-hand *p*-values are the results of paired *t*-tests between methods with *p*-values lower than 0.05 indicating a fixed bias. Blue lines represent results from a linear regression of differences and mean values of methods. In ideal situations, differences between methods should not be correlated with the magnitude of assessed parameters. Right-hand *p*-values indicate if there was a significant slope of the regression function with *p*-values lower than 0.05 indicating a proportional bias. Green lines indicate the upper and lower lines of agreement between methods and span an interval of two standard deviations above and below the mean difference. Outliers beyond lines of agreement render methods by definition not equivalent. Concerning RMT estimates, only MRI_warped_5_ and MRI_warped_36_ conditions yielded results that were equivalent to MRI_individual_ estimates.

RMT estimates from the MRI_warped_5_ and MRI_warped_36_ conditions were equivalent to MRI_individual_ estimates based on Bland-Altman plots and related criteria for methodological agreement (Figure 2). Mean differences to MRI_individual_ estimates were −0.05 ± 2.28% (*t*_(19)_ = −0.09, *p* = 0.923) and −0.1 ± 1.65% (*t*_(19)_ = −0.27, *p* = 0.789) maximum stimulator output (MSO) in MRI_warped_5_ and MRI_warped_36_ conditions, respectively. There was furthermore no outlier data in these conditions and the regression analysis revealed non-significant slopes of −0.08 (*t*_(18)_ = − 1.36, *p* = 0.189) and −0.05 (*t*_(18)_ = − 1.2, *p* = 0.244), respectively. Limits of agreement were narrower in the MRI_36_ as compared to the MRI_5_ condition given the lower standard deviation of differences. RMT estimates in the MRI_warped_9_ condition included outlier data and the regression analysis revealed a proportional bias with a significant slope of −0.15 (*t*_(18)_ = − 2.28, *p* = 0.035). The non-navigated condition was associated with a fixed bias and significantly over-estimated the RMT by 1.45 ± 2.84%MSO (*t*_(19)_ = 2.29, *p* = 0.034).

**Figure 3:**
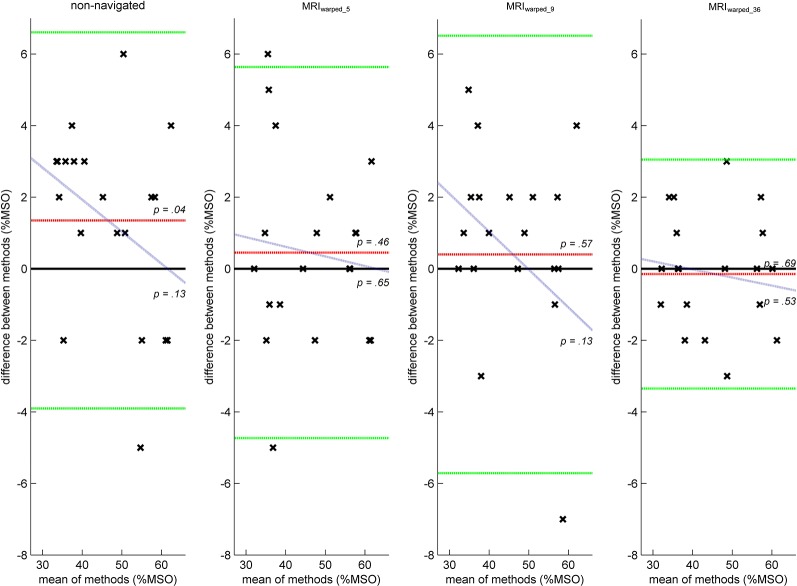
Bland-Altman plots illustrating the agreement of 500 μV motor threshold (500 μV-MT) estimates between conditions. Figure descriptions related to the construction of Bland-Altman plots and associated parameters are equal to detailed descriptions in Figure 2. Estimates of non-navigated conditions exhibited a significant fixed bias and overestimated 500 μV-MT by 1.35 ± 2.68%MSO (*t*_(19)_ = 2.25, *p* = 0.036). There was no proportional between MRI_individual_ estimates and either of the tested methods. However, there were outliers beyond lines of agreement in the non-navigated and MRI_warped_5_ and MRI_warped_9_ conditions rendering all methods inequivalent to the reference condition except for MRI_warped_36_ which agreed significantly.

500μV-MT estimates could only be considered equivalent between MRI_individual_ and MRI_warped_36_ conditions. There was neither a significant absolute difference between methods [−0.15 ± 1.63%MSO; (*t*_(19)_ = −0.41, *p* = 0.686) nor a proportional bias (slope −0.02; *t*_(18)_ = −0.65, *p* = 0.527)] or any data beyond limits of agreement (Figure 3). All other conditions included data beyond limits of agreement. The non-navigated condition also exhibited a significant fixed bias of 1.35 ± 2.68%MSO (*t*_(19)_ = 2.25, *p* = 0.036).

**Figure 4:**
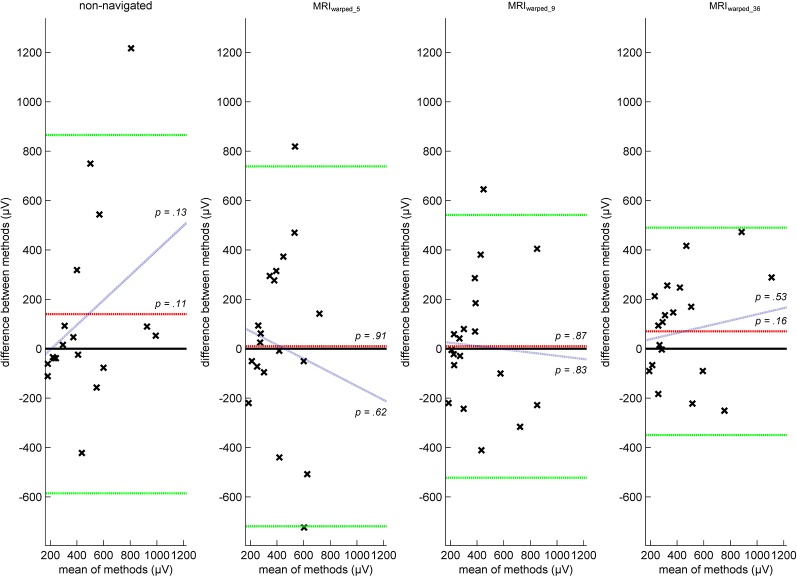
Bland-Altman plots illustrating the agreement of motor evoked potential (MEP) dispersion between conditions. Figure descriptions related to the construction of Bland-Altman plots and associated parameters are equal to detailed descriptions in Figure 2. MEP dispersion was quantified by the mean absolute difference of 30 consecutive MEP applied at 500 μV-MT intensity. MAD was chosen instead of standard deviation since it is a more robust measure of dispersion when underlying data is not normally distributed, which is well known for MEP data at least for an initial transient state of about 20 stimuli. There was no fixed or proportional bias between the reference condition (MRI_individual_) and either of the tested methods. Yet, all but the MRI_warped_36_ condition included data beyond limits of agreement rendering results inequivalent.

MEP variance examined at 500 μV-MT intensity was equivalent between MRI_individual_ and MRI_warped_36_. The MAD difference between methods was 70.5 ± 214.38 μV (*t*_(19)_ = 1.47, *p* = 0.158) with a non-significant slope of 0.12 (*t*_(18)_ = 0.64, *p* = 0.532) and did not include outlier data (Figure 4). All other conditions had wider limits of agreement but still included outlier data and are therefore not methodologically equivalent to MRI_individual_ results.

### Interrater Reliability Motor Thresholds

In line with the equivalence of topographical results as outlines above, estimates of motor thresholds also showed excellent inter-rater reliabilities. RMT estimates from MRI_individual_ conditions had an ICC of 0.951 (0.817–988), non-navigated conditions had an ICC of 0.878 (0.584–968), MRI_warped_5_ conditions had an ICC of 0.947 (0.801–986), MRI_warped_9_ had an ICC of 0.961 (0.853–990) and MRI_warped_36_ conditions had an ICC of 0.888 (0.616–971). Estimates of 500 μV-MT had an ICC of 948 (0.807–987) in MRI_individual_ conditions, 0.974 (0.899–993) in non-navigated conditions, 0.969 (0.879–992) in MRI_warped_5_ conditions, 0.954 (0.826–988) in MRI_warped_9_ conditions and 0.939 (0.776–985) in MRI_warped_36_ conditions.

## Discussion

This study aimed to test the hypothesis that nTMS examinations of M1 excitability yield equal topographical and functional results irrespective of the MRI type used for navigation. Results provide direct evidence that the notion of equivalence is true for hotspot locations, motor thresholds, and corticospinal excitability when a sufficient number of head surface coordinates are used to construct MRI_warped_.

### Feasibility and Accuracy of nTMS With Individualized Template MRI

The spatial error reported for the MRI warping procedure was lower than 3 mm throughout conditions and hence as low as that reported for spatial normalization procedures including affine (linear) and non-linear methods used in functional brain imaging (Grachev et al., 1999; Salmond et al., 2002). The fact that the least warping error was found in the condition with the least number of head surface coordinates is unexpected but indicates that the digitization of nearby, unlike remote coordinates, was more critically affected by inevitable minor system inaccuracies (Ruohonen and Karhu, 2010). The global mean error was nonetheless lower than that reported in the study by Carducci and Brusco (2012) who estimated a disagreement of 4.69 ± 2.21 mm.

MRI_warped_5_ and MRI_warped_9_ co-registrations were more inaccurate than MRI_individual_ registrations given the substantially higher number of rejected registrations. MRI image imperfections were reported to be the main source of error and to contribute with about 2.5 mm to the total system accuracy error (Ruohonen and Karhu, 2010). From the system’s co-registration display it became apparent that coordinates in the sagittal plane were in good agreement throughout while spatial errors increased with more lateral locations in MRI_warped_5_ and MRI_warped_9_ conditions. It is on retrospect indeed plausible that individualized template MRI constructed based on few registration points were scaled too short on the x-axis (SPM) since three out of five (MRI_warped_5_) or seven out of nine (MRI_warped_9_) coordinates passed to the optimization function were located around the sagittal plane. A local minimum of the warping error was thus likely to be found with a transformation matrix that favors an optimization of central coordinates.

### Spatial Consistency of Hotspots Between Navigation Conditions

Previous studies compared the intersession reliability of primary motor hotspots in TMS exams and provide important information on what can be expected in ideal conditions, i.e., when identical researchers repeatedly use nTMS based on equal and individual anatomic information. Wolf et al. (2004) reported that hotspots moved between 8.9 ± 4.6 mm (left hemisphere) and 10.5 ± 3.4 mm (right hemisphere) over three sessions that were 7–14 days apart. Zdunczyk et al. (2013) found that hotspots moved on average between 5.29 ± 2.02 mm and 6.63 ± 0.2.31 mm over sessions that were at least 1 week apart and performed by a novice and experienced examiner, respectively. Based on these studies, hotspot distances of about 5 mm found in the MRI_warped_36_ condition should be regarded as insignificant. Unexpectedly, distances to MRI_individual_ hotspots were shorter in the non-navigated condition than in MRI_warped_5_ and MRI_warped_9_ conditions. Spatial navigation and anatomical guidance inaccuracies may both partially account for this finding. Spatial navigation may have been flawed in these conditions by an insufficient agreement of the head model and actual head shape. Spatial disagreement may also have yielded erroneous anatomical information, which could have additionally impaired performance in MRI_warped_5_ and MRI_warped_9_ conditions rendering it inferior even to non-navigated conditions.

In summary, nTMS with accurate spatial navigation yields superior topographical results compared to non-navigated TMS irrespective of the availability of individual anatomical guidance. Yet, inaccurate spatial navigation and anatomical guidance may negatively affect nTMS examinations and even bias experienced users to select ill-defined hotspots despite clear instructions to rely on a functional hotspot definition.

### Functional Equivalence of TMS Parameters

Motor thresholds and MEP variance were equivalent between MRI_individual_ and MRI_warped_36_ conditions while significantly different results were obtained when nTMS with MRI_warped_ based on fewer head surface coordinates or non-navigated TMS was used. Yet, motor threshold estimates differed up to 3% MSO even in MRI_warped_36_ conditions which need to be discussed. The most intuitive explanation would be that at least some of the difference reflects physiological fluctuations of corticospinal excitability (Schmidt et al., 2009). Studies investigating trial-to-trial variability of corticospinal output in humans indicate that short-term fluctuations with a coefficient of variation up to 0.3 are to be expected (Burke et al., 1995). Residual disagreements and worse performance in other navigated conditions most likely reflects different hotspot locations. Additional influences by other systematic differences between conditions are unlikely given the cross-over design and that non-physiological stimulation parameters were equally well controlled in all navigation conditions. This being said, hotspot locations in non-navigated conditions agreed better with MRI_individual_ hotspot locations than MRI_warped_5_ and MRI_warped_9_ locations, yet functional results were equally poor. This discrepancy could be explained by the fact that the control for non-physiological stimulation parameters in MRI_warped_5_ and MRI_warped_9_ conditions partially compensated for spatial inaccuracy. It was indeed one of our secondary hypotheses that nTMS using MRI_warped_ would outperform non-navigated TMS.

### Interrater Reliability of Motor Thresholds

ICC were excellent throughout motor threshold estimates and navigation conditions. While mean values seem to indicate a difference between groups, it is important to consider that confidence intervals are largely overlapping rendering differences insignificant. To our knowledge, there is just one other study that investigated inter-rater reliabilities of motor thresholds in TMS studies (Fleischmann et al., 2013). Zdunczyk et al. (2013) compared resting motor thresholds (RMTs) assessed by a novice and an expert researcher in different sessions similar to our study design. They reported a median difference between RMT estimates of 0.5% MSO with a range of −6–15% MSO. The corresponding ICC was 0.63. Agreement between raters was hence significantly worse than in all conditions in our study although they used the same maximum-likelihood threshold-hunting algorithm that we used (Awiszus, 2003). An intuitive explanation would be that their hotspot locations varied more than in our study but they reported mean differences between hotspot locations of less than 5 mm which is close to the limit of the systems’ accuracy and can hardly be improved. Another possibility is that the less experienced researcher in our study was not a novice researcher as was the less experienced researcher in the study by Zdunczyk et al. (2013) but had about 1 year of experience with nTMS. This limited experience may have sufficed to be in better agreement with the expert researcher.

### Limitations

While we were able to prove the equivalence of nTMS using MRI_individual_ and MRI_warped_36_ based on statistical criteria, it remains unclear what the true difference between methods might be. This is because we did not include a condition that allows for an estimation of physiological fluctuations of TMS measures of cortical excitability, which was shown to confound topographical and functional TMS estimates even in navigated conditions (Schmidt et al., 2015). A repeated nTMS session using MRI_individual_ would be a suitable way to evaluate true deviations beyond physiological fluctuation.

Another limitation is that results from this study only apply to nTMS examinations of healthy individuals with unchanged cortical anatomy, but including older individuals with global atrophy that were shown not to exhibit major reorganization of their M1 topography (Yousry et al., 1997). While the hotspot would still have a functional definition in patients with structural changes including stroke and brain tumors, anatomical information is of paramount importance in these situations for several reasons. It was repeatedly shown that compensatory motor plasticity can be associated with shifts of motor hotspots to sensory and premotor locations which might be missed in cases of distorted anatomy without anatomical information (Fridman et al., 2004; Carey et al., 2006; Picht et al., 2011). Anatomical information is also essential for the presurgical mapping of corticospinal representations in tumor patients (Picht et al., 2011). Recently proposed predictive algorithms for post-stroke recovery furthermore include the assessment of preserved MEP responses in the affected hemisphere which can be judged with more certainty if correct stimulation locations can be verified (Stinear et al., 2012).

Results are furthermore clearly confined to the primary motor cortex since other cortical areas do not provide MEP as a simple read-out of the stimulation location. It might nonetheless be desirable to target other areas not using an individual MRI for similar reasons as addressed in this study. Applications in other areas would require validation using other read-outs such as reaction time or other (e.g., visual, TMS-) evoked potential paradigms.

The lack of true anatomical information will furthermore preclude the correlation of electrophysiological results with connectivity measures from functional or diffusion-weighted MRI. TMS studies also increasingly use electrical field strength instead of %MSO, which is not possible using individualized MRI since this approach requires knowledge about individual coil-cortex-distances (Schmidt et al., 2015).

## Conclusion

This is the first study to prove in direct comparison that topographical and functional results are equivalent between nTMS examinations with spatial navigation using individualized and individual MRI in the primary motor area of healthy individuals. Results provide evidence that the acquisition of individual MRI might be redundant for studies that perform single or repeated neurophysiological examinations over primary motor hotspots.

## Data Availability Statement

The datasets generated for this study are available on request to the corresponding author.

## Ethics Statement

The studies involving human participants were reviewed and approved by Ethikkommission der Charité – Universitätsmedizin Berlin. The patients/participants provided their written informed consent to participate in this study.

## Author Contributions

RF, AK, and SS conceived and planned the experiments. RF and AK carried out the experiments. All authors contributed to the interpretation of the results and provided critical feedback and helped shape the research, analysis, and manuscript. RF and SS took the lead in writing the manuscript.

## Conflict of Interest

The authors declare that the research was conducted in the absence of any commercial or financial relationships that could be construed as a potential conflict of interest. The reviewer TP declared a shared affiliation, with no collaboration, with the authors to the handling editor at the time of review.
